# Differential Effects of Genetically Determined Cholesterol Efflux Capacity on Coronary Artery Disease and Ischemic Stroke

**DOI:** 10.3389/fcvm.2022.891148

**Published:** 2022-07-04

**Authors:** Aoming Jin, Mengxing Wang, Weiqi Chen, Hongyi Yan, Xianglong Xiang, Yuesong Pan

**Affiliations:** ^1^Department of Neurology, Beijing Tiantan Hospital, Capital Medical University, Beijing, China; ^2^China National Clinical Research Center for Neurological Diseases, Beijing Tiantan Hospital, Capital Medical University, Beijing, China

**Keywords:** cholesterol efflux capacity, Mendelian randomization, coronary artery disease, stroke, genetics

## Abstract

**Background:**

Observational studies indicated that cholesterol efflux capacity (CEC) of high-density lipoprotein (HDL) is inversely associated with cardiovascular events, independently of the HDL cholesterol concentration. The aim of the study is to examine the casual relevance of CEC for coronary artery disease (CAD) and myocardial infarction (MI), and compare it with that for ischemic stroke and its subtypes using a Mendelian randomization approach.

**Methods:**

We performed a 2-sample Mendelian randomization to estimate the casual relationship of CEC with the risk of CAD, MI, and ischemic stroke. A CEC-related genetic variant (rs141622900) and other five genetic variants were used as the instrumental variables. Association of genetic variants with CAD were estimated in a GWAS involving 60,801 CAD cases and 123,504 controls. They were then compared with the associations of these variants with ischemic stroke and its subtypes (large vessel, small vessel, and cardioembolic) involving 40,585 ischemic stroke cases and 406,111 controls.

**Results:**

Using the SNP of rs141622900 as the instrument, a 1-SD increase in CEC was associated with 45% lower risk for CAD (odds ratio [OR] 0.55, 95% confidence interval [CI] 0.44–0.69, *p* < 0.001) and 33% lower risk for MI (odds ratio [OR] 0.67, 95% CI 0.52–0.87, *p* = 0.002). By contrast, the causal effect of CEC was much weaker for ischemic stroke (odds ratio [OR] 0.79, 95% CI 0.64–0.97, *p* = 0.02; *p* for heterogeneity = 0.03) and, in particular, for cardioembolic stroke (*p* for heterogeneity = 0.006) when compared with that for CAD. Results using five genetic variants as the instrument also indicated consistently weaker effects on ischemic stroke than on CAD.

**Conclusion:**

Genetic predicted higher CEC may be associated with decreased risk of CAD. However, the casual association of CEC with ischemic stroke and specific subtypes would need to be validated in further Mendelian randomization studies.

## Introduction

Epidemiologic studies have shown an inverse relationship between high-density lipoprotein (HDL) cholesterol levels and cardiovascular disease (1); however, recent clinical trials (2, 3) and Mendelian randomization (MR) studies (4, 5) failed to established a clear causal association between HDL cholesterol and cardiovascular disease. This led to the hypothesis that the atheroprotective role of HDL lies in its function rather than in its concentrations (6).

The most important measure of HDL function is cholesterol efflux capacity (CEC), the ability of HDL to reverse cholesterol transport from peripheral cells (7). Previous cohort and case-control studies showed that CEC was inversely associated with atherosclerosis and the incidence of cardiovascular events in the general population, independently of the HDL cholesterol concentration (8–11). However, observational epidemiological studies may suffer from confounding and selection bias that represent obstacles to valid causal inference (12, 13). The causal association between CEC and cardiovascular diseases is still controversial. Furthermore, ischemic stroke had a heterogeneous mechanism and may have different cause and risk factors from coronary artery disease (CAD) (14). Previous MR studies have showed a weaker effect on ischemic stroke than on CAD for some lipid metabolic factors, such as low-density lipoprotein cholesterol and proprotein convertase subtilisin/kexin type 9 (PCSK9) variants (15, 16). Therefore, the relative effects of CEC on CAD and ischemic stroke needs further investigation.

MR study, using genetic variants as instrumental variables, is a method that can control potential confounders and reverse causation that may bias observational studies, and make stronger causal inferences between an exposure and risk of diseases (12). In the present study, we aimed to use MR analysis to examine the causal relevance of CEC for CAD and myocardial infarction (MI), and compares it with that for ischemic stroke and its subtypes.

## Materials and Methods

### Study Design

A two-sample MR analysis using CEC-related genetic variants as instrumental variable was designed to evaluate the causal effect between CEC and risk of CAD and ischemic stroke (Supplementary Figure 1). Summary-level data on the exposure (CEC) were derived from a recent published genome-wide association study (GWAS) of up to 5,293 European individuals (17) and data on the outcome (CAD and ischemic stroke) were obtained from GWASs of up to 446,696 European individuals (18, 19). Table 1 and Supplementary Table 1 shows the characteristics of these GWASs. Approval of ethics committee and written informed consent were obtained before data collection in the original GWASs.

**Table 1:** Characteristics of the GWAS studies used in this study.

**Phenotype**	**Consortium**	***N***	**Ethnicity**	**Genotype data**	**PMID**
**Exposure**	
Cholesterol efflux capacity (CEC)		Up to 5,293 individuals	European	GWAS array	30369316
**Outcomes**	
Coronary artery disease (CAD)	CARDIoGRAMplusC4D	Up to 184,305 individuals	European	GWAS array	26343387
Myocardial infarction (MI)	CARDIoGRAMplusC4D	Up to 171,876 individuals	European	GWAS array	26343387
Ischemic stroke (IS)	MEGASTROKE	Up to 446,696 individuals	European	GWAS array	29531354
Large artery stroke (LAS)	MEGASTROKE	Up to 440,328 individuals	European	GWAS array	29531354
Small vessel stroke (SVS)	MEGASTROKE	Up to 411,497 individuals	European	GWAS array	29531354
Cardioembolic stroke (CES)	MEGASTROKE	Up to 413,304 individuals	European	GWAS array	29531354

### Genetic Instrumental Variables

We used 6 single nucleotide polymorphisms (SNPs) associated with CEC identified through GWAS by Low-Kam et al. (17) as the instrumental variables. Low-Kam et al. (17) tested the genetic association between 4 CEC measures and genotypes at >9 million common autosomal DNA sequence variants in 5,293 French Canadians. They identified 10 genome-wide significant signals (*P* < 6.25 × 10^−9^) representing 7 loci. Among the 7 loci, 2 loci (near the *PPP1CB/PLB1* and *RBFOX3/ENPP7* genes) only reached genome-wide significance in the model further adjusted for HDL-C and triglyceride levels which may lead to false positive associations in the GWAS context (i.e., collider bias). Other 5 loci (*CETP, LIPC, LPL, APOA1/C3/A4/A5*, and *APOE/C1/C2/C4*) harbored genes with important roles in lipid biology and reached genome-wide significance in the model adjusted for sex, age squared, coronary artery disease status, experimental batches, statin treatment, and the first 10 principal components. Except for the *APOE/C1/C2/C4* variant, association of other 4 loci disappeared when correcting for HDL-C and triglyceride levels. Only the SNP of rs141622900 in *APOE/C1/C2/C4* locus reached genome-wide significance in both two models and was used as the instrument. In sensitivity analysis, we used the most significant SNP in each of the 5 loci (rs77069344, rs2070895, rs247616, rs964184, and rs445925) as the instrument. These 5 SNPs were in different genomic regions and not in linkage disequilibrium (*r*^2^ <0.1). The 1 SNP (rs141622900) instrument explained 0.9% and the 5 SNPs instrument explained 5.3% of the variance in CEC (*F* statistic = 59.2 and 45.9, respectively, indicating sufficient strength of the instruments). Table 2 shows the characteristics and associations of these included SNPs with CEC.

**Table 2:** Characteristics of the included SNP loci associated with cholesterol efflux capacity.

**SNP**	**Locus**	**Chromosome (Position) (hg19)**	**EA/OA**	**EAF**	**CEC**	**Beta**	**SE**	***p***
rs77069344	*LPL*	8 (19 821 782)	G/T	0.099	J774 basal	0.2008	0.0327	7.96 × 10^−10^
rs2070895	*LIPC*	15 (58 723 939)	A/G	0.230	J774 basal	0.1424	0.0232	8.49 × 10^−10^
rs247616	*CETP*	16 (56 989 590)	T/C	0.314	J774 basal	0.1466	0.0211	4.08 × 10^−12^
rs964184	*APOA1/C3/A4/A5*	11 (116 648 917)	C/G	0.857	J774 ABCA1 dependent	0.2019	0.0281	6.78 × 10^−13^
rs445925	*APOE/C1/C2/C4*	19 (45 415 640)	A/G	0.114	J774 ABCA1 dependent	0.2155	0.0303	1.20 × 10^−12^
rs141622900	*APOE/C1/C2/C4*	19 (45 426 792)	A/G	0.058	BHK stimulated	0.2833	0.0417	1.03 × 10^−11^

*SNP, single nucleotide polymorphism; EA, effect allele; OA, other allele; EAF, effect allele frequency; CEC, cholesterol efflux capacity; SE, standard error. The unit of beta coefficients is SD increase of CEC per allele*.

### Outcomes

Summary statistics for the association of each CEC-related SNP with the CAD and MI were extracted from the Coronary ARtery DIsease Genome-wide Replication And Meta-Analysis Plus Coronary Artery Disease Genetics (CARDIoGRAMplusC4D) 1000 Genomes-based GWAS (17). The CARDIoGRAMplusC4D 1000 Genomes-based GWAS interrogated 9.4 million variants in up to 60,801 CAD cases and 123,504 controls from 48 studies of predominantly European ancestry. Summary statistics for the association of the included SNPs with ischemic stroke and the 3 main subtypes of ischemic stroke (large artery stroke [LAS], small vessel stroke [SVS], cardioembolic stroke [CES]) were extracted from the GWAS of Multiancestry Genome-wide Association Study of Stroke (MEGASTROKE) consortium (19). The MEGASTROKE consortium tested ~8 million SNPs and indels with minor-allele frequency ≥0.01 in up to 67,162 stroke cases and 454,450 controls from 29 studies, predominantly European ancestry (40,585 cases; 406,111 controls). This GWAS involved 34,217 cases with LAS, 5,386 cases with SVS and 7,193 cases with CES of European ancestry. The associations of the 6 individual SNPs for CEC with CAD and MI, and ischemic stroke and its subtypes are presented in Tables 3, 4, respectively.

**Table 3:** Genetic association of cholesterol efflux capacity related genetic variants with coronary artery disease and myocardial infarction in the CARDIoGRAMplusC4D consortium.

**SNPs**	**EA/OA**	**Coronary artery disease**			**Myocardial infarction**		
		**Beta**	**SE**	***p***	**Beta**	**SE**	***p***
rs77069344	G/T	−0.0514	0.0158	0.001	−0.0651	0.0176	0.000
rs2070895	A/G	0.0372	0.0108	0.001	0.0414	0.0121	0.001
rs247616	T/C	−0.0312	0.0103	0.002	−0.0280	0.0114	0.014
rs964184	C/G	−0.0500	0.0124	0.000	−0.0488	0.0139	0.000
rs445925	A/G	−0.0858	0.0187	0.000	−0.0664	0.0214	0.002
rs141622900	A/G	−0.1421	0.0278	0.000	−0.0963	0.0315	0.002

EA, effect allele; OA, other allele; SE, standard error; SNP, single nucleotide polymorphism. The unit of beta coefficients is log-odds per allele. The odds ratio = exp(Beta), upper bound of odds ratio = exp(Beta+1.96^*^SE), and lower bound of odds ratio = exp(Beta-1.96^*^SE).

**Table 4:** Genetic association of cholesterol efflux capacity related genetic variants with ischemic stroke and its subtypes in the MEGASTROKE consortium.

**SNPs**	**EA/OA**	**Ischemic stroke**			**LAS**			**SVS**			**CES**		
		**Beta**	**SE**	***p***	**Beta**	**SE**	***p***	**Beta**	**SE**	***P***	**Beta**	**SE**	***p***
rs77069344	G/T	0.0153	0.0160	0.339	0.0450	0.0396	0.256	0.0062	0.0371	0.868	0.0207	0.0316	0.514
rs2070895	A/G	−0.0033	0.0121	0.783	−0.0587	0.0304	0.054	0.0367	0.0278	0.187	0.0136	0.0235	0.563
rs247616	T/C	0.0082	0.0110	0.455	−0.0168	0.0276	0.542	−0.0119	0.0258	0.645	0.0108	0.0212	0.609
rs964184	C/G	0.0181	0.0152	0.233	0.0060	0.0373	0.872	−0.0071	0.0349	0.838	0.0122	0.0297	0.681
rs445925	A/G	−0.0298	0.0184	0.106	−0.0723	0.0461	0.117	−0.0365	0.0413	0.378	−0.0362	0.0350	0.301
rs141622900	A/G	−0.0579	0.0254	0.023	−0.0963	0.0679	0.156	−0.0793	0.0598	0.185	0.0178	0.0509	0.727

EA, effect allele; OA, other allele; LAS, large artery stroke; SVS, small vessel stroke; CES, cardioembolic stroke; SE, standard error; SNP, single nucleotide polymorphism. The unit of beta coefficients is log-odds per allele. The odds ratio = exp(Beta), upper bound of odds ratio = exp(Beta+1.96^*^SE), and lower bound of odds ratio = exp(Beta-1.96^*^SE).

### Statistical Analysis

Per-allele effects of the selected SNPs on CEC and disease outcomes were extracted from the GWASs and used to estimate the causal effect of CEC on outcomes using two-sample MR analyses. Using the SNP of rs141622900 as the instrument, Wald ratio method were used to obtain effect estimate by dividing the SNP-outcome estimate by the SNP-CEC estimate. Standard error were estimated using the Delta method by dividing the SNP-outcome standard error by the SNP-CEC estimate (20). When using the 5 SNPs as the instruments, we used a conventional inverse-variance weighted (IVW) MR analysis with multiplicative random effects assuming all genetic variants are valid instruments. In IVW method, the SNP-outcome estimate is regressed on the SNP-CEC estimate, weighted by the inverse-variance of SNP-outcome estimate and with the y-axis intercept is fixed to zero (21). We further conducted methodologic sensitivity analyses using MR-Egger, simple median, weighted median methods using the 5 SNPs as the instruments, which are more robust to the inclusion of pleiotropic or invalid instruments. MR-Egger method can assess and control for directional pleiotropic bias and provide an pleiotropy-corrected effect estimate in which genetic variants are permitted to be invalid instrumental variables (22). The median methods can provide a consistent effect estimate using the median of the empirical distribution function of individual SNP ratio estimates in which up to 50% of the genetic variants are permitted to be invalid instruments (23). Presences of heterogeneity between causal effects of individual variants and comparisons between the causal effects of CEC on CAD vs. ischemic stroke were tested using the Cochran Q statistic and *I*^2^ index in the IVW analysis (23). Evidence of pleiotropic effects were assessed using intercepts of the MR-Egger regression (22). Moreover, multivariable two-sample MR were performed to adjust for major causes of survival (smoking, body mass index, and blood pressure) using the 5 SNPs as the instruments (24). Multivariable MR analysis was used to assess whether the associations between genetic predisposition to CEC and ischemic stroke may be affected by selection bias (25). The above analysis were conducted in the UK Biobank GWAS and FinnGen GWAS as sensitivity analyses.

The percentage of variance explained in CEC was estimated by 2 × (effect on CEC)^2^ × minor allele frequency × (1- minor allele frequency) (16). A power analysis was performed using a web-based application (https://sb452.shinyapps.io/power/). Effect estimates of CEC-outcome (CAD, MI, ischemic stroke and its subtypes) are presented as odds ratios (ORs) with their 95% confidence intervals (CIs) of outcome per 1-SD genetically higher CEC. To account for multiple testing, a Bonferroni-corrected significance level of *p* < 0.0083 (i.e., 0.05/6 for 6 outcomes) was predefined as statistically significant evidence for a causal association. All analyses were conducted with R 3.5.1 (R Development Core Team).

## Results

**Figure 1:**
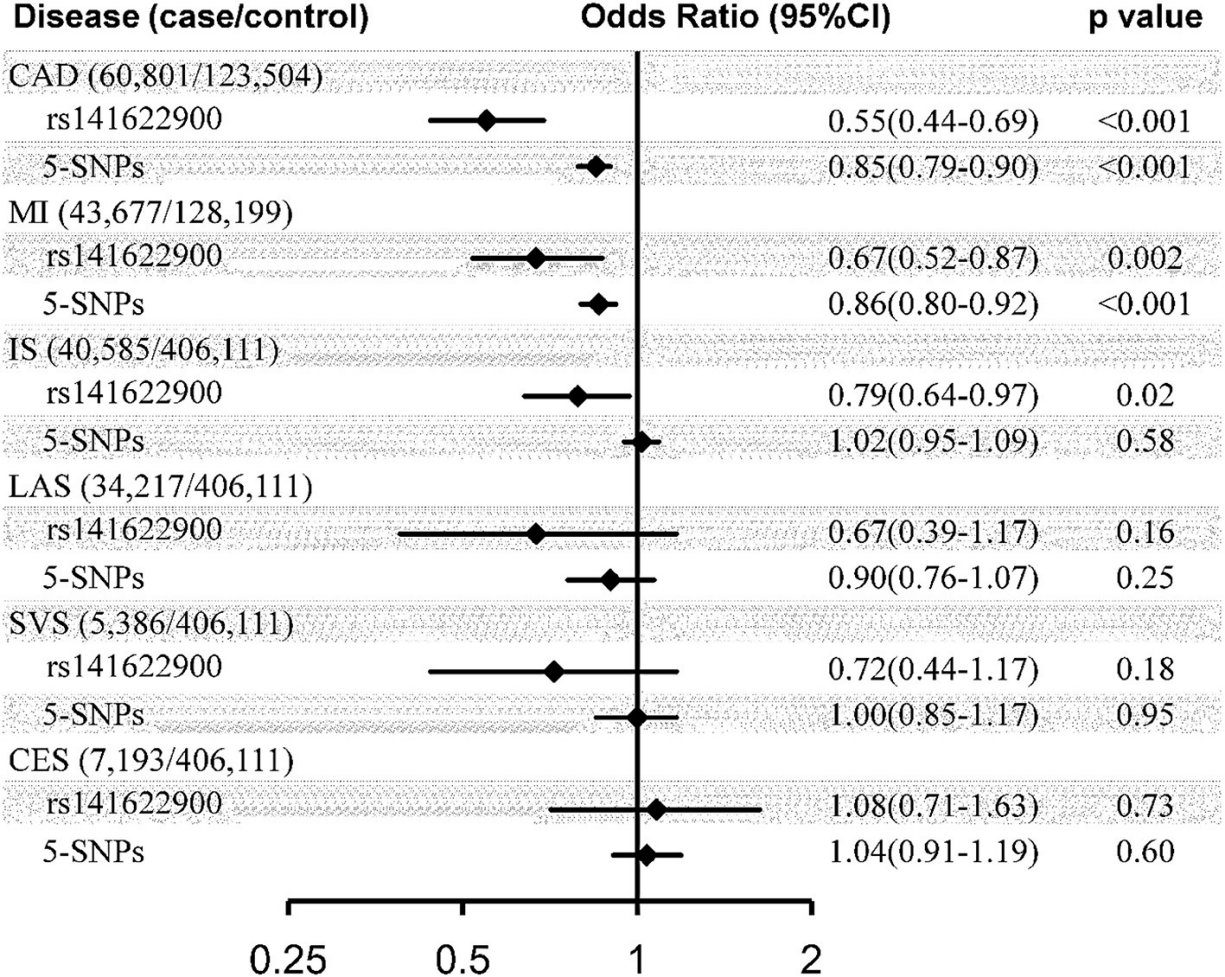
Causal effect estimates of genetically predicted cholesterol efflux capacity on coronary artery disease and stroke. Estimates represented odds ratio (95% CI) per SD genetically higher cholesterol efflux capacity derived from Wald ratio method using rs141622900 as the instrument and inverse-variance weighted method using 5-SNPs (rs77069344, rs2070895, rs247616, rs964184, and rs445925) as the instrument. CI, confidence interval; SNP, single nucleotide polymorphism; CAD, coronary artery disease; MI, myocardial infarction; IS, ischemic stroke; LAS, large artery stroke; SVS, small vessel stroke; CES, cardioembolic stroke.

Genetically determined 1-SD increase in CEC was casually associated with a substantial decrease in risk of CAD (OR = 0.55, 95% CI: 0.44–0.69, *p* < 0.001) and MI (OR = 0.67, 95% CI: 0.52–0.87, *p* = 0.002); but, by contrast, was not causally associated with ischemic stroke (OR = 0.79, 95% CI: 0.64–0.97, *p* = 0.02) or any separate subtype of ischemic stroke (LAS: OR = 0.67, 95% CI: 0.39–1.17, *p* = 0.16; SVS: OR = 1.08, 95% CI: 0.71–1.63, *p* = 0.73; CES: OR = 0.72, 95% CI: 0.44–1.17, *p* = 0.18) at the Bonferroni-adjusted level of significance (*p* < 0.0083) using the SNP of rs141622900 as the instrument (Figure 1). The effect of CEC on ischemic stroke was weaker than that on CAD (*p* for heterogeneity = 0.03, *I*^2^ = 80%), and in particular on CES (*p* for heterogeneity = 0.006, *I*^2^ = 87%), whereas the effects of CEC on LAS and SVS were compatible with the magnitude of the effect observed for CAD (*p* for heterogeneity = 0.53 and 0.34, respectively). The effects of CEC on ischemic stroke and its subtypes were compatible with the magnitude of the effect for MI (*p* for heterogeneity = 0.34, 1.00, 0.80, and 0.06, respectively). These analyses had a >99, >99, 70, and 82% power to detect a 30% decrease in risk of ischemic stroke, LAS, SVS, and CES (equivalent to the upper limit of the CI for CAD), respectively; this can exclude a causal effect of CEC on ischemic stroke and CES of the same magnitude as on CAD, and indicate comparable effects of CEC on LAS, SVS, and CAD. Whereas, the power to detect a 13% decrease in risk of ischemic stroke, LAS, SVS, and CES (equivalent to the upper limit of the CI for MI) was 72, 65, 16, and 20%, respectively; this indicated comparatively little power for comparable effects of CEC on ischemic stroke, particular stroke subtypes and MI.

Similar disparate associations of CEC were observed with the risk of CAD (OR = 0.85, 95% CI: 0.79–0.90, *p* < 0.001) and MI (OR = 0.86, 95% CI: 0.80–0.92, *p* < 0.001), compared to ischemic stroke (OR = 1.02, 95% CI: 0.95–1.09, *p* = 0.58) and its subtypes (LAS: OR = 0.90, 95% CI: 0.76–1.07, *p* = 0.25; SVS: OR = 1.00, 95% CI: 0.85–1.17, *p* = 0.95; CES: OR = 1.04, 95% CI: 0.91–1.19, *p* = 0.60), using the IVW methods with the 5-SNPs instrument (Figure 1). The effect of CEC on ischemic stroke was weaker than that on CAD (*p* for heterogeneity <0.001, *I*^2^ = 93%) and MI (p for heterogeneity <0.001, *I*^2^ = 91%), and in particular on CES (*p* for heterogeneity = 0.008, *I*^2^ = 86%; p for heterogeneity = 0.01, *I*^2^ = 83%), whereas the effects of CEC on LAS and SVS were compatible with the magnitude of the effect observed for CAD (*p* for heterogeneity = 0.49 and 0.07, respectively) and MI (*p* for heterogeneity = 0.58 and 0.10, respectively). These analyses had a >99, 99, 42, and 53% power to detect a 10% decrease in risk of ischemic stroke, LAS, SVS, and CES (equivalent to the upper limit of the CI for CAD and MI), respectively; this can exclude a causal effect of CEC on ischemic stroke of the same magnitude as on CAD and MI, and indicate comparable effects of CEC on LAS, CAD, and MI.

Significant association for CAD and MI and insignificant association for ischemic stroke and its subtypes were also observed in sensitivity analyses using the MR-Egger, simple median and weighted median methods using the 5-SNPs instrument (Table 5). Evidence of heterogeneity was observed for the outcome of CAD and MI (*Q* = 42.5, *p* < 0.001; *Q* = 35.4, *p* < 0.001) but not for the outcome of ischemic stroke or its subtypes (*Q* = 5.3, *p* = 0.26; *Q* = 6.6, *p* = 0.16; *Q* = 2.8, *p* = 0.59; *Q* = 2.0, *p* = 0.74) in the IVW analysis. MR-Egger regression showed no evidence of directional pleiotropy for the association of CEC with all disease outcomes (all *p* value for intercept >0.05) (Table 5). In sensitivity analyses using FinnGen GWAS data, significant associations for ischemic heart disease and MI and insignificant association for ischemic stroke and CES were observed using the SNP of rs141622900 as the instrument. However, no significant associations for the outcomes was observed in the IVW analysis using the 5-SNPs instrument (Supplementary Table 2). In the multivariable MR adjusting for major causes of survival using the 5-SNPs instrument, the associations of CEC with ischemic stroke and its subtypes remained insignificant, which were similar to the estimates by the IVW method (Supplementary Table 3). Similar results of insignificant associations for ischemic stroke were observed in the UK Biobank data (Supplementary Table 4).

**Table 5:** MR statistical sensitivity analyses using 5 SNPs as the instrumental variables^*^.

**Outcome**	**MR-Egger**				**Simple median**		**Weighted median**	
	**OR (95% CI)**	***P***	**Intercept (95% CI)**	***p* value for intercept**	**OR (95% CI)**	***P***	**OR (95% CI)**	***p***
CAD (60,801/123,504)	0.35 (0.14–0.89)	0.03	0.156 (−0.007, 0.319)	0.06	0.78 (0.71–0.86)	<0.001	0.79 (0.71–0.87)	<0.001
MI (43,677/128,199)	0.34 (0.13–0.89)	0.03	0.161 (−0.004, 0.326)	0.056	0.79 (0.70–0.88)	<0.001	0.79 (0.71–0.88)	<0.001
IS (40,585/406,111)	0.97 (0.57–1.65)	0.92	0.008 (−0.083, 0.100)	0.86	1.06 (0.96–1.17)	0.26	1.06 (0.97–1.16)	0.21
LAS (34,217/406,111)	1.60 (0.43–6.00)	0.49	−0.101 (−0.330, 0.128)	0.39	0.89 (0.69–1.16)	0.39	0.92 (0.72–1.17)	0.49
SVS (5,386/406,111)	0.66 (0.26–1.64)	0.37	0.074 (−0.086, 0.233)	0.37	0.97 (0.78–1.19)	0.74	0.96 (0.78–1.19)	0.72
CES (7,193/406,111)	0.79 (0.36–1.71)	0.55	0.048 (−0.086, 0.182)	0.48	1.08 (0.90–1.28)	0.41	1.08 (0.91–1.28)	0.41

MR, mendelian randomization; OR, odds ratio; CI, confidence interval; SNP, single nucleotide polymorphism; CAD, coronary artery disease; MI, myocardial infarction; IS, ischemic stroke; LAS, large artery stroke; SVS, small vessel stroke; CES, cardioembolic stroke. *Five SNPs in the instrument included rs77069344, rs2070895, rs247616, rs964184, and rs445925.

## Discussion

The present study is the first large-scale assessment and comparison of causal relevance of CEC and the risk of vascular disease using Mendelian randomization approach. The results showed that genetic predicted higher CEC may be associated with decreased risk of CAD. However, the casual association of CEC with ischemic stroke and specific subtypes would need to be validated in further Mendelian randomization studies.

Our findings of inverse relationship between CEC and CAD and MI are consistent with several meta-analysis summarizing previous observational studies (10, 26, 27). Although results from the majority of studies were in line with the hypothesis that higher CEC is associated with lower risk of CAD (9, 11, 28–31), a study by Li showed that increased HDL-mediated CEC was paradoxically associated with increased risk for incident myocardial infarction or stroke, which based on the study population undergoing coronary angiography (32). Moreover, the German Diabetes Dialysis Study (4D Study) failed to observe an significant association of CEC with the composite outcome (cardiac death, nonfatal MI, and stroke) in patients with end-stage renal disease (33). The CEC was quantified using human THP-1-derived macrophage foam cells loaded with cholesterol, which was different from cAMP (cyclic adenosine monophosphate)-stimulated murine J774 macrophages employed by other studies in the general population (8, 11). The reasons for the apparent discrepancies among previous studies in the relationship between CEC and CAD are unclear but could be ascribed to difference in sample size, study population, study design, and methods for CEC measurements across studies. Considering the heterogeneity between observational studies and potential confounders that warrant caution, MR studies using genetic variants as instrumental variables could provide more robust evidence for the causal relationship of CEC and the health outcome of interest. The present study showed genetic predicted higher CEC was associated with lower CAD risk, which supports the direct causal association between CEC and CAD.

Our study does not support a causal role of CEC in ischemic stroke. Few studies have investigated the association between CEC and ischemic stroke and its subtypes expect two cohort studies with inconsistent results (9, 29). Results from the MESA (Multi-Ethnic Study of Atherosclerosis) cohort showed no relationship of cholesterol mass efflux capacity with stroke or with non-hemorrhagic stroke. However, a small subgroup (*n* = 37) of the Dallas Heart study reported an inverse association between CEC and stroke. Using genetic variants related to CEC as the instrument, the association between CEC and ischemic stroke was examined directly in our study. However, we found no evidence of significant causal relationships between CEC and ischemic stroke and its subtypes. In the present Mendelian randomization study, the estimates of CEC with ischemic stroke might be biased by sample selection on surviving exposure of interest and on surviving competing risk of the outcome (24). However, the results of multivariable MR analyses conducted by adjusting for potential causes of survival were consistent with the main results. As with any selection bias correction by multivariable adjustment, it may not be feasible to recover the valid estimate. We repeated the analyses in the UK Biobank data and FinnGen data, respectively. Both results were similar to the main results in the present study. Larger scale Mendelian randomization studies are still needed to clarify the genetic effects of CEC on ischemic stroke and assess any heterogeneity between ischemic stroke subtypes, which may shed light on the relationship of CEC on ischemic stroke further. Furthermore, in these future studies, the effects of genetically determined CEC on ischemic stroke could be compared between patients with vascular cognitive impairment vs. patients with non-cognitive impairment.

The HDL-mediated CEC is the ability to remove excess cholesterol from lipid-laden macrophages representing the first crucial step within the process of reverse cholesterol transport (7). Reverse cholesterol transport plays an important role in atheroprotective mechanism by facilitating the removal of cholesterol in the arterial wall and the subsequent decrease in the proinflammatory response (34, 35). Our study found that the effect of CEC was weaker on ischemic stroke than CAD, which were consistently observed in such comparisons in the effects of other blood lipid on vascular disease in recent Mendelian studies. A Mendelian study suggested that the effects of LDL cholesterol on ischemic stroke was weaker than that on coronary heart disease (16). Moreover, another Mendelian study showed PCSK9 genetic variants had smaller associations with risk of ischemic stroke than with risk of coronary heart disease (15). The potential explanation for the difference between the effects of CEC on CAD and ischemic stroke is the biological differences in these disease process. Ischemic stroke involves phenotypic heterogeneity, with different biological pathways for LAS, SVS, and CES, compared to the more homogenous CAD phenotype (36). Moreover, a review reported that hematological disorders were the most frequent etiology of cerebral infarcts of unusual cause (37). Except for the usual cerebrovascular risk factors such as hypertension, diabetes mellitus, and dyslipidemia, other newly factors could be considered of the causal relevance for ischemic stroke. Furthermore, differences in the distribution of risk factors as well as patient characteristics between CARDIoGRAMPlusC4D and METASTROKE consortium may partly explain the different effects for CAD and IS observed in the study. Additionally, insufficient statistical power due to small sample size, especially for stroke subtypes, may be considered as another reason. Anacetrapib, an CETP (cholesteryl ester transfer protein) inhibitor that was developed for increasing HDL cholesterol levels and promoting CEC to a greater degree, was shown a significant reduction in CAD in the REVEAL trial (38). Although it met its primary endpoint, the small improvement against the main goal and the safety of CETP inhibitor had become a point of contention consistently. Finally, Anacetrapib was not filed for approval with the US Food and Drug Administration. Our study provided supporting evidence for the causal relationship between CEC and risk of CAD, indicating potential intervention targets to the increase of CEC for improving cardiovascular outcomes.

The present Mendelian randomization analyses relies on three underlying assumptions. First, we identified 6 CEC-related SNPs (*P* < 6.25 × 10^−9^) served as instruments in the MR analysis that satisfied the first assumption. Second, we did not find that the 5-SNPs in the study were associated with other key lipids including low-density lipoprotein, triglycerides, total cholesterol, and apolipoprotein B based on GWAS datasets. And the results of multivariable MR analyses and sensitivity analyses using the UK Biobank data and FinnGen data were consistent with the main results. Thus, these results increase confidence in the validity of the second assumption that there is no confounding (measured or unmeasured) of the genetic variants with the outcome. Third, all the genetic variants were not directly associated with the outcomes (all *P* > 5 × 10^−8^), which suggested that the third assumption was not violated.

Our study has several limitations. First, the study was conducted based on datasets of predominantly European ancestry and generalization of the results to other populations of non-European ancestry was limited. However, the uniformity of the included subjects may minimize the risk of bias by population admixture. Second, the sample sizes of ischemic stroke subtypes were still relatively small, specifically for SVS and CES. Insignificant association between CEC and ischemic stroke subtypes could be attributed to insufficient statistical power. However, most estimates were consistent using different MR approaches, which suggests that the observed associations are not by chance. Third, although the GWAS that we used to identify all CEC-related SNPs represents the first and largest effort to identify genome-wide significant loci associated with CEC, sample size remains modest (*N* = 5,293 participants), and therefore there is a limitation of power to find weak effect variants. In the main analysis, only one SNP (rs141622900) was used as the instrument. The SNP is strongly associated with many key lipids relevant to cardiovascular disease, such as low-density lipoprotein and apolipoprotein B. The pleiotropic effects of the CEC-related SNPs on other key lipids was unable to be assessed by two-sample multivariable MR in the present study because of the lack of data. However, we have conducted MR-Egger regression using 5 CEC-related SNPs as the instrumental variables to assess evidence of pleiotropic effects in the study. Though the results showed no evidence of directional pleiotropy, further studies are needed to validate the associations of CEC with disease outcomes. Forth, we performed multivariable MR and sensitivity analyses to correct selection bias. However, recovering the valid estimates of CEC for ischemic stroke has not been fully addressed. Finally, Mendelian randomization has been considered as an alternative approach for causal inferences with a lot of advantages compared to randomized controlled trials. However, it cannot replace randomized controlled trials in establishing a claim of causality (39). Future clinical trials are still needed with sufficient statistical power to validate the causal relationship of CEC.

The study examined causal relationships between CEC and risk of vascular disease using MR analysis, and suggests that genetic predicted higher CEC may be associated with decreased risk of CAD. However, the casual association of CEC with ischemic stroke and specific subtypes would need to be validated in further Mendelian randomization studies.

## Data Availability Statement

The original contributions presented in the study are included in the article/Supplementary Material, further inquiries can be directed to the corresponding authors.

## Ethics Statement

Ethical review and approval was not required for the study on human participants in accordance with the local legislation and institutional requirements. Written informed consent for participation was not required for this study in accordance with the national legislation and the institutional requirements.

## Author Contributions

AJ and MW performed the study, analyzed the data, wrote the paper, and reviewed drafts of the paper. WC and XX wrote the paper and reviewed drafts of the paper. HY analyzed the data, prepared figures and tables, and reviewed drafts of the paper. YP conceived and designed the study, performed the study, analyzed the data, wrote the paper, and reviewed drafts of the paper. All authors contributed to the article and approved the submitted version.

## Funding

This work was supported by grants from the National Natural Science Foundation of China (81971091 and 81901177), Beijing Hospitals Authority Youth Programme (QML20190501 and QML20200501), Young Elite Scientist Sponsorship Program from China Association for Science and Technology (2019QNRC001), Beijing Tiantan Hospital, Capital Medical University (2018-YQN-1 and 2020MP01) and Outstanding Young Talents Project of Capital Medical University (A2105).

## Conflict of Interest

The authors declare that the research was conducted in the absence of any commercial or financial relationships that could be construed as a potential conflict of interest.

## Publisher's Note

All claims expressed in this article are solely those of the authors and do not necessarily represent those of their affiliated organizations, or those of the publisher, the editors and the reviewers. Any product that may be evaluated in this article, or claim that may be made by its manufacturer, is not guaranteed or endorsed by the publisher.
