# Supplementary material for: Differential Effects of Genetically Determined Cholesterol Efflux Capacity on Coronary Artery Disease and Ischemic Stroke
Source: Front Cardiovasc Med. 2022 Jul 4;9:891148. doi: 10.3389/fcvm.2022.891148 (PMC9289203; doi:10.3389/fcvm.2022.891148)
Supplement: Supplementary file 1 [file Data_Sheet_1.pdf]

Supplementary Table 1. Study details for the GWAS of the CAD, MI, and ischemic stroke.

Consortium	Cases	Control	Mean age	Sex	Phenotype	Phenotype definition	Adjusted variables
Cardiogram 1000 genomes GWAS	60,801	123,504	NA, possibly ~58 years	NA	CAD	Several different definitions including electronic databases, ICD codes, et al	Study-specific Covariates (not age or sex)
MEGASTROKE	40,585	406,111	~69 years	NA	IS	Several different definitions including electronic databases, ICD codes, et al	Minimum of age and sex

CAD, coronary artery disease; IS, ischemic stroke.

Supplementary Table 2. MR statistical sensitivity analyses using FinnGen GWAS*.

Outcome	Genetic instrument variable			MR-Egger		Simple median		Weighted median	
		OR (95% CI)	P value	OR (95% CI)	P value	OR (95% CI)	P value	OR (95% CI)	p value
IHD	rs141622900	0.58(0.48-0.71)	<0.001						
	5 SNP*	0.78(0.58-1.06)	0.11	0.17(0.10-0.29)	0.02	0.65(0.56-0.76)	0.01	0.74(0.64-0.85)	<0.001
MI	rs141622900	0.46(0.35-0.61)	<0.001						
	5 SNP*	0.75(0.51-1.1)	0.14	0.11(0.06-0.22)	0.02	0.61(0.5-0.74)	0.02	0.69(0.58-0.81)	<0.001
IS	rs141622900	1.1(0.84-1.43)	0.48						
	5 SNP*	1.1(0.95-1.27)	0.07	1.37(0.52-3.62)	0.59	1.02(0.86-1.22)	0.81	1.03(0.9-1.18)	0.67
CES	rs141622900	0.87(0.33-2.34)	0.79						
	5 SNP*	0.9(0.56-1.46)	0.25	0.22(0.01-3.75)	0.41	1.33(0.55-3.23)	0.57	0.89(0.56-1.43)	0.64

MR, mendelian randomization; OR, odds ratio; CI, confidence interval; SNP, single nucleotide polymorphism; IHD, ischemic heart disease; MI, myocardial infarction; IS, ischemic stroke; CES, cardioembolic stroke. * Five SNPs in the instrument included rs77069344, rs2070895, rs247616, rs964184 and rs445925.

Supplementary Table 3. Association of genetic predisposition to cholesterol efflux capacity with ischemic stroke and its subtypes in univariable and multivariable inverse-variance weighted analyses*.

Outcome	Cases	Univariable IVW		MVMR adjusted for BMI[a]		MVMR adjusted for smoking[b]		MVMR adjusted for SBP[c]		MVMR adjusted for DBP[d]	
		OR (95% CI)	P value	OR (95% CI)	P value	OR (95% CI)	P value	OR (95% CI)	P value	OR (95% CI)	P value
IS	40,585	0.97(0.57-1.65)	0.92	1.03(0.94, 1.13)	0.54	1.01(0.91, 1.13)	0.82	1.04(0.95, 1.14)	0.48	1.01(0.91, 1.13)	0.84
LAS	34,217	1.60(0.43-6.00)	0.49	0.88(0.69, 1.14)	0.41	0.85(0.66, 1.10)	0.30	0.95(0.75, 1.22)	0.73	0.91(0.68, 1.23)	0.60
SVS	5,386	0.66(0.26-1.64)	0.37	1.02(0.89, 1.17)	0.81	1.02(0.86, 1.21)	0.83	1.04(0.91, 1.19)	0.62	1.04(0.89, 1.21)	0.64
CES	7,193	0.79(0.36-1.71)	0.55	1.06(0.97, 1.15)	0.32	1.03(0.91, 1.17)	0.64	1.07(0.98, 1.17)	0.22	1.05(0.92, 1.19)	0.52

MVMR, multivariable mendelian randomization; IVW, inverse-variance weighted; BMI, body mass index; SBP, systolic blood pressure; DBP, diastolic blood pressure; OR, odds ratio; CI, confidence interval; SNP, single nucleotide polymorphism; IS, ischemic stroke; LAS, large artery stroke; SVS, small vessel stroke; CES, cardioembolic stroke.

* The instrumental variables included five SNPs (rs77069344, rs2070895, rs247616, rs964184 and rs445925).

a Summary-level data for BMI were obtained from Pulit et al, Hum Mol Genet 2019, PMID 30239722.

b Summary-level data for smoking were obtained from Marcus Munafo, Wootton (2019): Genome-wide association study of lifetime smoking index in a sample of 462,690 individuals from the UK Biobank.

c Summary-level data for SBP were obtained from Evangelou Eet al, Nat Genet 2018, PMI: 30224653.

d Summary-level data for DBP were obtained from Evangelou Eet al, Nat Genet 2018, PMI: 30224653.

Supplementary Table 4. MR statistical sensitivity analyses using UK Biobank GWAS*.

Outcome	Genetic instrument variable			MR-Egger		Simple median		Weighted median	
		OR (95% CI)	P value	OR (95% CI)	P value	OR (95% CI)	P value	OR (95% CI)	p value
IS	rs141622900	0.44 (0.07-2.75)	0.38						
	5 SNP*	1.19(0.55-2.60)	0.66	0.21(0.002-17.29)	0.54	1.33(0.35-5.01)	0.69	1.33(0.38-4.72)	0.68

IS, ischemic stroke. * Five SNPs in the instrument included rs77069344, rs2070895, rs247616, rs964184 and rs445925.

Supplementary Figure 1. Conceptual framework for the Mendelian randomization analysis of cholesterol efflux capacity and risk of coronary artery disease and stroke. SNP, single nucleotide polymorphism.

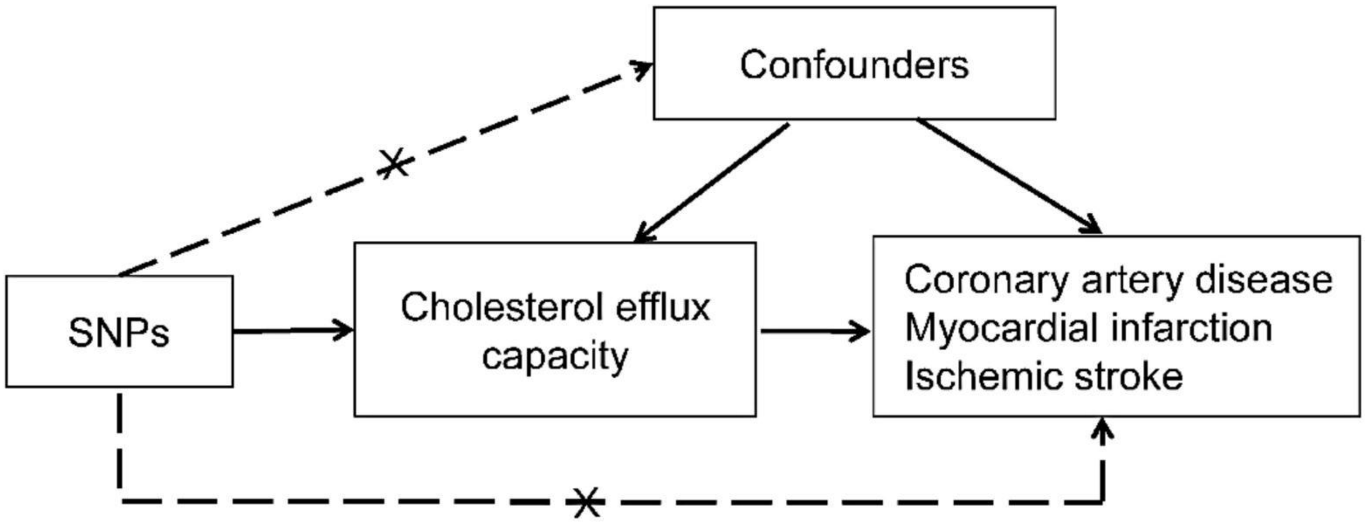

STROBE-MR checklist of recommended items to address in reports of Mendelian randomization studies[1 2]

Item No.	Section	Checklist item	Page No.	Relevant text from manuscript
1	**TITLE and ABSTRACT**	Indicate Mendelian randomization (MR) as the study's design in the title and/or the abstract if that is a main purpose of the study	Title Page and Page 1-2	
	**INTRODUCTION**			
2	**Background**	Explain the scientific background and rationale for the reported study. What is the exposure? Is a potential causal relationship between exposure and outcome plausible? Justify why MR is a helpful method to address the study question	Page 3	1st and 2nd paragraph
3	**Objectives**	State specific objectives clearly, including pre-specified causal hypotheses (if any). State that MR is a method that, under specific assumptions, intends to estimate causal effects	Page 3	3rd paragraph
	**METHODS**			
4	**Study design and data sources**	Present key elements of the study design early in the article. Consider including a table listing sources of data for all phases of the study. For each data source contributing to the analysis, describe the following:	Page 4	Table 1 and Table S1 in Supplement
	a)	Setting: Describe the study design and the underlying population, if	Page 4,5	Table 1

			possible. Describe the setting, locations, and relevant dates, including periods of recruitment, exposure, follow-up, and data collection, when available.		
		b)	Participants: Give the eligibility criteria, and the sources and methods of selection of participants. Report the sample size, and whether any power or sample size calculations were carried out prior to the main analysis	Page 5	2nd paragraph, Table S1 in Supplement
		c)	Describe measurement, quality control and selection of genetic variants	Page 4,5	Table 2
		d)	For each exposure, outcome, and other relevant variables, describe methods of assessment and diagnostic criteria for diseases	Page 4,5	Table 3,4
		e)	Provide details of ethics committee approval and participant informed consent, if relevant	Page 4	1st paragraph
5	**Assumptions**		Explicitly state the three core IV assumptions for the main analysis (relevance, independence and exclusion restriction) as well assumptions for any additional or sensitivity analysis	Page 4	Figure S1 in Supplement
6	**Statistical methods: main analysis**		Describe statistical methods and statistics used	Page 6,7	
		a)	Describe how quantitative variables were handled in the analyses (i.e., scale, units, model)	Page 6	1st paragraph

		b)	Describe how genetic variants were handled in the analyses and, if applicable, how their weights were selected	Page 6	1st paragraph
		c)	Describe the MR estimator (e.g. two-stage least squares, Wald ratio) and related statistics. Detail the included covariates and, in case of two-sample MR, whether the same covariate set was used for adjustment in the two samples	Page 6	1st paragraph
		d)	Explain how missing data were addressed	NA	
		e)	If applicable, indicate how multiple testing was addressed	Page 7	2nd paragraph
7	**Assessment of assumptions**		Describe any methods or prior knowledge used to assess the assumptions or justify their validity	Page 6,7	1st paragraph
8	**Sensitivity analyses and additional analyses**		Describe any sensitivity analyses or additional analyses performed (e.g. comparison of effect estimates from different approaches, independent replication, bias analytic techniques, validation of instruments, simulations)	Page 6,7	1st paragraph and 2nd paragraph
9	**Software and pre-registration**				
		a)	Name statistical software and package(s), including version and settings used	Page 7	2nd paragraph
		b)	State whether the study protocol and details were pre-registered (as well as	NA	

			when and where)		
	**RESULTS**				
10	**Descriptive data**				
		a)	Report the numbers of individuals at each stage of included studies and reasons for exclusion. Consider use of a flow diagram	Page 4, Table 1	Table 1
		b)	Report summary statistics for phenotypic exposure(s), outcome(s), and other relevant variables (e.g. means, SDs, proportions)	Table 2	Table 2
		c)	If the data sources include meta-analyses of previous studies, provide the assessments of heterogeneity across these studies	NA	
		d)	For two-sample MR:<br>i. Provide justification of the similarity of the genetic variant-exposure associations between the exposure and outcome samples<br>ii. Provide information on the number of individuals who overlap between the exposure and outcome studies	Page 4	Table 1 and Table S1 in Supplement
11	**Main results**				
		a)	Report the associations between genetic variant and exposure, and between genetic variant and outcome, preferably on an interpretable scale	Table 2,3,4	Table 2,3,4
		b)	Report MR estimates of the relationship between exposure and outcome,	Page 7,8	Table 5

			and the measures of uncertainty from the MR analysis, on an interpretable scale, such as odds ratio or relative risk per SD difference		
		c)	If relevant, consider translating estimates of relative risk into absolute risk for a meaningful time period	NA	
		d)	Consider plots to visualize results (e.g. forest plot, scatterplot of associations between genetic variants and outcome versus between genetic variants and exposure)	Figure 1	Figure 1
12	**Assessment of assumptions**				
		a)	Report the assessment of the validity of the assumptions	Page 8,9	Table 2, 1st paragraph
		b)	Report any additional statistics (e.g., assessments of heterogeneity across genetic variants, such as I^2, Q statistic or E-value)	Page 7,8,9	
13	**Sensitivity analyses and additional analyses**				
		a)	Report any sensitivity analyses to assess the robustness of the main results to violations of the assumptions	Page 8,9	1st paragraph, Table 5
		b)	Report results from other sensitivity analyses or additional analyses	Page 8,9	

		c)	Report any assessment of direction of causal relationship (e.g., bidirectional MR)	NA	
		d)	When relevant, report and compare with estimates from non-MR analyses	Page 9	3rd paragraph
		e)	Consider additional plots to visualize results (e.g., leave-one-out analyses)	NA	
	**DISCUSSION**				
14	**Key results**		Summarize key results with reference to study objectives	Page 9	2nd paragraph
15	**Limitations**		Discuss limitations of the study, taking into account the validity of the IV assumptions, other sources of potential bias, and imprecision. Discuss both direction and magnitude of any potential bias and any efforts to address them	Page 13	1st paragraph
16	**Interpretation**				
		a)	Meaning: Give a cautious overall interpretation of results in the context of their limitations and in comparison with other studies	Page 9,10	
		b)	Mechanism: Discuss underlying biological mechanisms that could drive a potential causal relationship between the investigated exposure and the outcome, and whether the gene-environment equivalence assumption is reasonable. Use causal language carefully, clarifying that IV estimates may provide causal effects only under certain assumptions	Page 11	2nd paragraph

		c) Clinical relevance: Discuss whether the results have clinical or public policy relevance, and to what extent they inform effect sizes of possible interventions	Page 12	1st paragraph
17	**Generalizability**	Discuss the generalizability of the study results (a) to other populations, (b) across other exposure periods/timings, and (c) across other levels of exposure	Page 13	1st paragraph
	**OTHER INFORMATION**			
18	**Funding**	Describe sources of funding and the role of funders in the present study and, if applicable, sources of funding for the databases and original study or studies on which the present study is based	Page 14	Sources of Funding
19	**Data and data sharing**	Provide the data used to perform all analyses or report where and how the data can be accessed, and reference these sources in the article. Provide the statistical code needed to reproduce the results in the article, or report whether the code is publicly accessible and if so, where	Page 14	Availability of data and materials
20	**Conflicts of Interest**	All authors should declare all potential conflicts of interest	Page 14	Disclosures

This checklist is copyrighted by the Equator Network under the Creative Commons Attribution 3.0 Unported (CC BY 3.0) license.

1. Skrivankova VW, Richmond RC, Woolf BAR, Yarmolinsky J, Davies NM, Swanson SA, et al. Strengthening the Reporting of Observational Studies in Epidemiology using Mendelian Randomization (STROBE-MR) Statement. JAMA. 2021;under review.

2. Skrivankova VW, Richmond RC, Woolf BAR, Davies NM, Swanson SA, VanderWeele TJ, et al. Strengthening the Reporting of Observational Studies in Epidemiology using Mendelian Randomisation (STROBE-MR): Explanation and Elaboration. BMJ. 2021;375:n2233.
